# A case of spontaneous abdominal hemoperitoneum secondary to ruptured splenosis

**DOI:** 10.1093/jscr/rjae284

**Published:** 2024-05-07

**Authors:** Akshata Gunda, Mary P Martos, Erin M Dickey, Alan S Livingstone, Caitlin Hester

**Affiliations:** Department of Medical Education, Miller School of Medicine, University of Miami, Miami, FL 33101, United States; Department of Surgery, Miller School of Medicine, University of Miami, Miami, FL 33136, United States; Department of Surgery, Miller School of Medicine, University of Miami, Miami, FL 33136, United States; Division of Surgical Oncology, Department of Surgery, Sylvester Comprehensive Cancer Center, Miller School of Medicine, University of Miami, Miami, FL 33136, United States; Division of Surgical Oncology, Department of Surgery, Sylvester Comprehensive Cancer Center, Miller School of Medicine, University of Miami, Miami, FL 33136, United States

**Keywords:** splenosis, spontaneous, hemoperitoneum, hemorrhage, splenectomy, complication

## Abstract

We present a case of spontaneous abdominal hemoperitoneum secondary to ruptured splenosis in a 35-year-old patient with a history of splenectomy secondary to trauma 23 years prior. Computed tomography imaging demonstrated a large amorphous mass-like structure in the mesentery of the left hemiabdomen with active extravasation and hemoperitoneum. The patient also had a separate focus of hyper-enhancing mass adjacent to the bladder representing a mass versus splenule. The patient’s radiographic and clinical presentation prompted management with exploratory laparotomy, hematoma evacuation, and resection of two splenules. With only a few cases of spontaneous abdominal hemoperitoneum from splenosis reported, this case describes successful management with surgical intervention.

## Introduction

Spontaneous hemorrhage from splenosis is a rare phenomenon that remains a subject of limited clinical documentation. While predominantly an asymptomatic, incidental finding, the available literature describes a few cases of spontaneous rupture of ectopic splenic tissue [1–5]. In this report, we present a compelling case of spontaneous hemorrhage from splenosis in a previously splenectomized patient presenting with acute massive abdominal hemoperitoneum, successfully managed with surgical intervention.

## Case presentation

**Figure 1:**
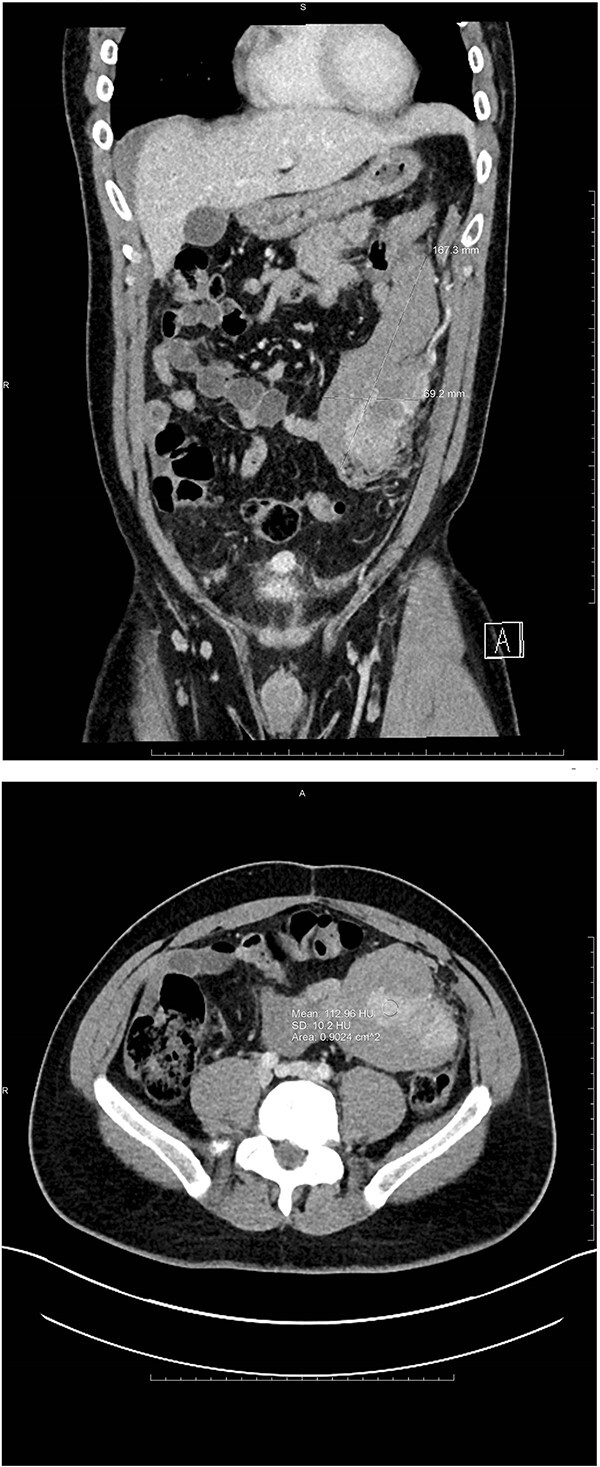
Coronal and axial views of CTA images of the abdomen and pelvis reveal a multilobulated heterogenous mass measuring 16.7 × 6.9 × 8.3 cm in the left mid-abdomen. Curvilinear foci of enhancement are visualized at its lateral aspect, originating from a prominent arterial vessel extending from the splenic artery.

A 35-year-old male presented to the emergency department with a 1-day history of worsening left abdominal pain after reported consumption of psilocybin mushrooms. The patient denied any other inciting events or trauma. He had a history of an exploratory laparotomy and splenectomy at 12 years of age due to a traumatic sports injury and was incidentally diagnosed with splenosis in his left abdomen and pelvis at an outside hospital during a workup for abdominal pain 6 years prior to presentation. Examination revealed tenderness in the left hemiabdomen with guarding as well as hemodynamics within the normal range. Point-of-care ultrasound demonstrated fluid in Morrison’s pouch as well as a large volume of fluid in the pelvis. A computed tomography (CT) scan with IV contrast showed a large amorphous 17-cm mass-like structure in the mesentery of his left hemiabdomen consistent with a hematoma, and a multilobulated heterogeneously enhancing lesion within the lateral aspect of this hematoma. Also noted was active extravasation at the lateral aspect of the mass, arising from a vessel that appeared to be a terminal branch of the main splenic artery but that terminated into the mass within the left abdomen/pelvis (Fig. 1).

**Figure 2:**
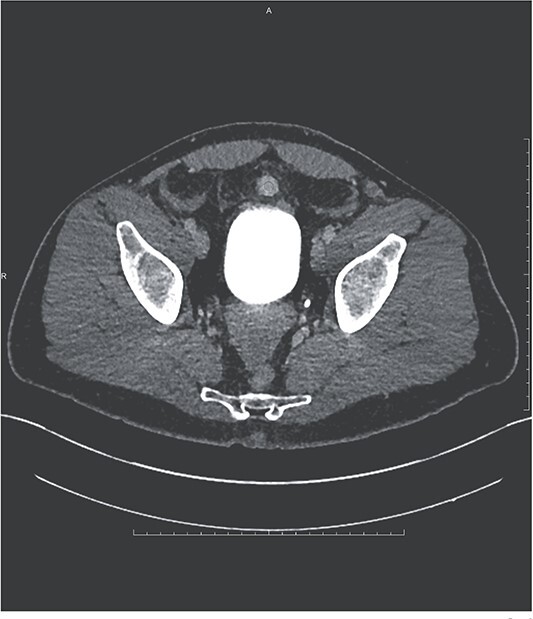
Ovoid hyperenhancing lesion anterior to the dome of the bladder, representing an additional area of splenosis.

Additional findings included hemorrhagic fluid in the pelvis, right paracolic gutter, and surrounding the right hepatic lobe as well as a small hyper-enhancing mass adjacent to the bladder (see Fig. 2).

The patient had a downtrending hemoglobin with a decrease from 13.1 to 11.5 g/dl over the course of 12 h. Surgical oncology was consulted due to concern for a bleeding tumor. Given his history and imaging findings, the team was less concerned with a malignancy and felt this more likely represented bleeding ectopic splenic tissue. The patient was a poor candidate for splenic embolization given the small caliber of the terminal inferior splenic artery and its length within the left lower abdomen. Given the active extravasation and a dropping hemoglobin, the decision was made to proceed with exploratory laparotomy.

Exploratory laparotomy revealed a large hematoma of ~600 ml arising from a splenule in the left abdomen and extending to the left upper quadrant. A feeding artery and vein were seen laterally and superiorly to the hematoma. The hematoma was evacuated, the splenule was dissected and freed from the surrounding bowel, and the feeding vessels were ligated. An additional splenule was found superior to the bladder and was resected. Surgical pathology demonstrated benign splenic tissue consistent with splenule with subcapsular and intraparenchymal hemorrhage and an associated 13 × 6 × 5 cm hematoma, as well as a separate pelvic splenule. The patient had an uneventful postoperative recovery apart from a delayed return of bowel function that did not require intervention.

## Discussion

Splenosis is thought to occur through the seeding of fragments of splenic tissue throughout a cavity during traumatic or iatrogenic compromise of the splenic capsule, with implants recruiting blood supply from local tissues and vessels to form nodules [6, 7]. Another hypothesis offers an alternative mechanism of hematogenous spread and seeding of splenic tissue [8]. Splenosis is a separate entity from the accessory spleen. An accessory spleen, often an incidental finding during splenectomies, is a congenital process and represents the failure of fusion of mesenchymal buds [9]. An accessory spleen is pathologically similar to normal splenic tissue in structure and function, while splenosis lacks a true hilum or splenic capsule and has less elastic and smooth muscle tissue [9, 10]. Additionally, location is a differentiating factor between the two pathologies, with an accessory spleen typically seen on the left side of the abdomen near the hilum of the spleen, and in proximity to the embryologic dorsal mesogastrium [5]. Splenosis, in contrast, can be found anywhere in the abdominal cavity; however, it is most often found dispersed in the peritoneal cavity along the omentum, peritoneum, and mesentery [10]. This patient’s history of traumatic splenectomy and the presence of distinct foci of splenic tissue in the left abdomen and pelvis support the diagnosis.

Despite the paucity of data on splenosis, it is recognized as a benign process with infrequent complications. There are rare reports of complications, such as small bowel obstruction, gastrointestinal hemorrhage, and pain due to infarction [10]. Acute hemorrhage from spontaneous rupture of splenosis is rare, with only a handful of cases documented [1–5]. In the current case report, the presentation was acute intraabdominal hemoperitoneum arising from a mass rather than gastrointestinal bleeding, making it one of only four other cases of acute spontaneous hemoperitoneum due to aberrant splenic tissue reported [1–4]. 75% of the prior cases were treated with surgical intervention [2].

The diagnosis and evaluation of intraperitoneal bleeding from splenosis can be a challenging clinical scenario. The differential diagnosis for splenosis includes tumor burden, metastases, lymphoma, vascular malformations, accessory spleen, and endometriosis in women [11, 12]. Following the diagnosis, the treatment will be case-dependent. Although this patient was a poor candidate for transarterial embolization, this treatment modality should be considered as an additional treatment option, with two such cases reported previously [13, 14]. In a few cases of hemorrhage from splenosis, conservative management has also been reported to be successful [2, 12, 15]. Of all the cases of splenosis and its varying complications, the literature on splenosis prevention is lacking. What remains imperative is a thorough history to identify past splenic trauma, to recognize splenosis as a differential diagnosis, and to manage the patient based on the clinical presentation.
